# Aliphatic extractive effects on acetic acid catalysis of typical agricultural residues to xylo-oligosaccharide and enzymatic hydrolyzability of cellulose

**DOI:** 10.1186/s13068-021-01952-8

**Published:** 2021-04-17

**Authors:** Jianming Guo, Kaixuan Huang, Rou Cao, Junhua Zhang, Yong Xu

**Affiliations:** 1 Key Laboratory of Forestry Genetics & Biotechnology, Ministry of Education, College of Chemical Engineering, Nanjing Forestry University, Nanjing, 210037 People’s Republic of China; 2 Jiangsu Co-Innovation Center of Efficient Processing and Utilization of Forest Resources, College of Chemical Engineering, Nanjing Forestry University, Nanjing, 210037 People’s Republic of China; 3 Jiangsu Province Key Laboratory of Green Biomass-Based Fuels and Chemicals, Nanjing, 210037 People’s Republic of China; 4 College of Forestry, Northwest A&F University, 3 Taicheng Road, Yangling, 712100 Shanxi, People’s Republic of China

**Keywords:** Aliphatic extractives, Acetic acid acidolysis, Xylo-oligosaccharides, Enzymatic hydrolysis, Agricultural residues

## Abstract

**Background:**

Xylo-oligosaccharide is the spotlight of functional sugar that improves the economic benefits of lignocellulose biorefinery. Acetic acid acidolysis technology provides a promising application for xylo-oligosaccharide commercial production, but it is restricted by the aliphatic (wax-like) compounds, which cover the outer and inner surfaces of plants.

**Results:**

We removed aliphatic compounds by extraction with two organic solvents. The benzene–ethanol extraction increased the yield of acidolyzed xylo-oligosaccharides of corncob, sugarcane bagasse, wheat straw, and poplar sawdust by 14.79, 21.05, 16.68, and 7.26% while ethanol extraction increased it by 11.88, 17.43, 1.26, and 13.64%, respectively.

**Conclusion:**

The single ethanol extraction was safer, more environmentally friendly, and more cost-effective than benzene–ethanol solvent. In short, organic solvent extraction provided a promising auxiliary method for the selective acidolysis of herbaceous xylan to xylo-oligosaccharides, while it had minimal impact on woody poplar.

## Background

Agricultural residues and forestry wood residues are some of the most abundant renewable resources in the world and can be obtained on a large scale at a low cost. The use of carbohydrates, which are present in agricultural residues, to produce biofuels and chemicals are both energy-efficient and environment-friendly [1]. Lignocellulosic materials such as agricultural wastes are composed of 40–50% cellulose, 25–30% hemicellulose, and 15–20% lignin along with other extractable components [2]. Among these constituents, cellulose and hemicellulose are macromolecules composed of different monosaccharides, mainly glucan and xylan, respectively, and are the main sugar platforms for biorefinery of, agricultural residues. In agricultural residues cellulose, hemicellulose, and lignin are bound through both covalent cross-linking and non-covalent forces. Lignin and hemicellulose wrap themselves around cellulose to form a watertight structure, resulting in a small accessible surface area, which makes the bioconversion of agricultural residues difficult [3].

Pretreatment of agricultural residues can break its natural structure either by dissolving or separating cellulose, hemicellulose, and lignin, thereby increasing its digestibility, resulting in effective bioconversion of cellulose and hemicellulose [4, 5]. In the past, several economic and effective pretreatment techniques have been used for lignocellulosic biorefining, resulting in oligosaccharides and monosaccharides. Up to now, the most concerned agricultural residues pretreatment techniques include steam explosion, dilute acid pretreatment, alkali pretreatment, ionic liquid pretreatment, and inorganic salt pretreatment [6, 7]. It is well known that pretreatment under mild conditions can selectively depolymerize the xylan skeleton in hemicellulose, producing xylo-oligosaccharides (XOS) as the main degradation product [8, 9]. Meanwhile, the removal of extractives and side reactions leading to monosaccharide degradation and lignin depolymerization will also occur in the reaction medium [10]. Our previous work found that the pretreatment of corncob (CC) [11], viscose fiber [12], and poplar [13] with green, mild, and recyclable acetic acid, resulted in effective depolymerization of hemicellulose and produced several xylan derivatives with high added value, such as XOS, xylose, and furfural. Thus, the combined conversion of hemicellulose and cellulose was performed through pretreatment with acetic acid combined with cellulase hydrolysis technology. Currently, this is a highly effective technique to achieve lignocellulosic biorefining.

XOS contains approximately 2–10 xylose units along with β-1,4-glycosidic bonds. It is also identified as emerging prebiotic products for human and animal use, feeding on stimulated intestinal bacteria, such as *Bifidobacterium*, *lactobacillus*, etc. [14, 15]. XOS is a novel functional ingredient that can be used in the fields of medicine, health care and feed, with a market price of $22–50/kg [16]. The conversion of hemicellulose xylan to XOS products is cost-effective and is critical for the commercialization of lignocellulosic biorefining. Therefore, there is extensive ongoing research on the synthesis of XOS. Acetic acid pretreatment is a green and economical hemicellulose degradation method [11, 17]. Zhang et al. obtained the highest XOS yield of 45.91% by pretreating CC with acetic acid, while achieving a cellulose conversion rate of more than 91%, thus achieving the efficient conversion of hemicellulose and cellulose [11]. However, pretreatment of wheat straw (WS), sugarcane bagasse (SB) and poplar sawdust (PS) with acetic acid only achieved the highest XOS yields of 38.21% [18], 39.1% [19] and 36.0% [20]. This discrepancy in yield might be attributed to a variety of reasons, including the low xylan content, high lignin content, and recalcitrant structure of wood capillary. These factors combine and enhance the differences of XOS preparations. Besides, it has been reported that the plant surface is covered with a protective layer of wax [21], which could have resulted in an inconsistent erosion and penetration of acetic acid into various agricultural and forestry wastes. The wax mentioned here refers to the cuticle on the plant surface, which is an epidermal lipid component covered on the outer epidermis of the aerial part of the plant. This cuticular wax regulates the moisture content inside the plant and prevents the infiltration of exogenous factors into the plant, thus acting as the structural stabilizing component of the primary epidermal tissue of the plant [22–24]. Many studies have shown that the removal of plant cuticular wax has some positive impacts on agricultural residues biorefinery. For example, Kádár et al. removed the cuticle and epidermis wax of WS through plasma-assisted pretreatment to increase the ethanol yield of WS from 21 to 67% [25]; Gao et al. dewaxed bagasse with the mixture of petroleum ether and ethanol also effectively improved the digestibility of cellulose and xylan of bagasse [26]. Therefore, dewaxing is a promising candidate technique to improve the yield of acidolyzed XOS from agricultural residues. The components of plant cuticle wax were mostly complex mixtures of long-chain aliphatic and a few were cyclic components [27, 28], and dewaxing methods include organic solvents extraction [22], supercritical carbon dioxide extraction [27, 29], alkali washing [30] and enzyme treatment [31], etc., wherein organic solvent extraction is the most popular technique for industrial plant dewaxing. Generally, volatile polar or non-polar organic solvents, such as methanol, ethanol, hexane, benzene, chloroform, petroleum ether and so on, are used for wax extraction. Benzene and ethanol are common extraction reagents. Therefore, in this study, benzene–ethanol mixed solvent and a single ethanol solvent were used to extract and compare agricultural residues, to obtain a more environmentally friendly and efficient wax removal method.

Here, four typical agricultural residues: CC, SB, WS, and PS were subjected to benzene–ethanol extraction (BEE) and ethanol extraction (EE) (preliminary dewaxing treatment), respectively, to verify the effect of removing the extractives on agricultural residues conversion, wherein poplar was set as a contrast sample opposite to grass agricultural residues. Next, we analyzed and compared the extracted components and explored the effects of preliminary dewaxing treatment on the yield of XOS after acetic acid catalysis and the enzymatic hydrolyzability of cellulose. Thus, this work proposed a composite pretreatment program that could effectively promote the high-value utilization of agricultural residues.

## Results and discussion

### Comparison of aliphatic extractives from various agricultural residues

The yield (based on dry matter) of extractives from CC, SB, WS, and PS from BEE was 1.60, 1.55, 2.07, and 1.22%, respectively, while from EE was 4.28, 2.71, 6.57, and 1.41%, respectively (Table 1). Generally, the yield obtained by EE was significantly higher than that of BEE. Among these four agricultural residues, the extraction yields of the total solids from CC, SB, and WS are markedly higher than that of woody PS, which was attributed to the fact that poplar logs have been peeled and processed compared with the intact herbages.

**Table 1:** Extracted solid and carbohydrate compounds of various agricultural residues

Materials	Extraction yield of total solid		Carbohydrate compounds of extractives detected by NREL-HPLC			
	BEE/%	EE/%	In the benzene–ethanol extractives		In the ethanol extractives	
			Glucose/%	Xylose/%	Glucose/%	Xylose/%
CC	1.60	4.28	1.38 ± 0.04	1.42 ± 0.01	4.33 ± 0.25	4.98 ± 0.37
SB	1.55	2.71	1.93 ± 0.32	4.50 ± 0.85	4.23 ± 0.25	6.50 ± 0.62
WS	2.07	6.57	0.86 ± 0.04	4.65 ± 0.28	3.05 ± 0.02	5.34 ± 0.14
PS	1.22	1.41	2.09 ± 0.34	0.85 ± 0.19	3.87 ± 0.26	3.10 ± 0.32

Based on the NREL method's composition analysis, we found that only glucose- and xylose-based compounds were present in various extractives, which accounted for approximately 3% of the total extractive mass. These extractable carbohydrates involved mainly oligosaccharides and trace amounts of monosaccharides, which were precursors of starch and cellulose. Unlike CC, SB, and WS, we found more glucose-based compounds than xylose-based compounds in the benzene–ethanol extractives and the ethanol extractives of PS. We speculated that was attributed to the presence of flavonoids in PS [32, 33], which could easily combine with sugar to form glycosides. After NREL acidolysis, glycosides generated glucose-like structures. Both BEE and EE were considered reasonable methods for the extraction of mainly aliphatic compounds other than detectable carbohydrate compounds from agricultural residues. The aliphatic extractives of woody poplar were obviously less than that of herbaceous materials. The ethanol–water solution extracted approximately 2–3 times more substrate than the benzene–ethanol solution since the former solution had higher polarity than the latter, which benefited the solubility of small and polar carbohydrates. On the contrary, benzene enhanced the non-polar nature of the extraction solvent that repelled polar sugars and compounds.

The composition of the non-derivatized solvent extractives was identified by GC–MS. Table 2 lists the fingerprints of the benzene–ethanol extractives and ethanol extractives of all four agricultural residues. The cuticular wax of plants mainly contains long-chain aliphatic compounds, derived from long-chain fatty acids, along with terpenes, flavonoids, sterols, etc. [34]. Aliphatic compounds were also the main extractives in BEE and EE from CC, SB, WS, and PS. From benzene–ethanol extractives and ethanol extractives of CC, we obtained cis-vaccenic acid and 2-palmitoylglycerol as the most abundant components, respectively. However, benzene–ethanol extractives of SB, WS, and PS revealed n-hexadecanoic acid as the most abundant component. The proportions of various compounds in the ethanol extractives of SB, WS, and PS were equivalent and contained a complex array of aliphatic compounds. Among these complex extractives of the four agricultural residues, n-hexadecanoic acid appeared as a common component, abundantly present in both the benzene–ethanol extractives and ethanol extractives, consistent with previous reports [34, 35]. Among the other components, n-hexadecanoic acid was a saturated fatty acid, cis-vaccenic acid was an unsaturated fatty acid, and 2-palmitoylglycerol was a fatty acid ester. These long-chain fatty acids are synthesized in the epidermis of plants and used for the formation of cuticular wax [34]. Cutin is a covalently cross-linked polymer that forms a dense electron layer on the epidermal cells, restraining plant growth and effectively resisting the attack of foreign impurities [36, 37].

**Table 2:** Composition and abundance of solvent extractives from various agricultural residues by GC–MS detection

Compounds		Area (%)							
		Benzene–ethanol extractives				Ethanol extractives			
		CC	SB	WS	PS	CC	SB	WS	PS
*n*-Fatty acids	Tetradecanoic acid			3.17	2.08				
	Pentadecanoic acid	1.58	1.31		2.81				
	N-Hexadecanoic acid	34.25	57.22	23.09	44.86	3.62	7.18	9.81	4.21
	Palmitoleic acid	4.50	7.14						
	Gamolenic acid				2.19				
	Heptadecanoic acid				1.33				
	9,12-Octadecadienoic acid		19.18		26.06				
	Cis-Vaccenic acid	37.98							
	Cis-13-Octadecenoic acid			1.88					
	Oleic acid	2.36		2.56					
	Octadecanoic acid		2.13		5.68			1.98	
*n*-Fatty alcohols	Phytol			3.11					
*n*-Alkanes	4-Methyl-decane						2.01	1.66	1.61
	2,6-Dimethyl-undecane						1.74		
	Dodecane			3.06			2.24	2.86	2.73
	4,6-Dimethyl-dodecane						2.71		2.05
	2,6,11-Trimethyl-dodecane						7.56	3.63	5.40
	2,6,10-Trimethyl-dodecane					2.68	1.72	3.38	6.05
	2,7,10-Trimethyl-dodecane						3.22		
	Tridecane			4.85					
	Tetradecane							3.35	
	Pentadecane						5.78		2.80
	4-Methyl-pentadecane								1.67
	Hexadecane							7.48	
	2,6,10,14-Tetramethyl-hexadecane						5.93		5.01
	2,6,11,15-Tetramethyl-hexadecane							3.29	4.02
	2,6,10,15-Tetramethyl-heptadecane						3.22	2.14	3.02
	Heptacosane					10.55	5.25	4.34	
	Octadecane								5.56
	Nonadecane							3.39	
	Heneicosane						5.90		
	Tetracosane						2.33		
	2-Methyl-hexacosane							2.59	2.19
	Hentriacontane					5.36			
Aldehydes	9,17-octadecadienal				1.96				
	13-Octadecenal			4.81					
	Vanillin	2.44						2.39	
Ketones	6,10,14-Trimethyl-2-pentadecanone			11.74					
	2-Heptadecanone			6.92					
Steroid ketones	6-Hydroxy-4,4,7a-trimethyl-5,6,7,7a-tetrahydrobenzofuran-2(4 h)-one							1.78	
	Dehydrovomifoliol							4.88	
	1-(4-Hydroxy-3,5-dimethoxyphenyl)-ethanone							3.23	
	4,5,6-Trimethoxy-7-methyl-3 h-2-benzofuran-1-one						2.44		
	2,3-Dihydro-benzofuran	3.34	6.59					1.74	
Sterols	4-(2,6,6-Trimethyl-1-cyclohexen-1-yl)-3-buten-2-ol						4.15		
	2-Methyl-4-(1,3,3-trimethyl-7-oxabicyclo [4. 1. 0] hept-2-yl)-3-buten-2-ol							2.44	
	3-Deoxyestradiol								1.60
Phenols	2-Methoxy-4-vinylphenol	2.45						2.06	
	2,4-Di-tert-butylphenol			3.52			2.50	2.06	2.21
	4-((1e)-3-Hydroxy-1-propenyl)-2-methoxyphenol	1.50	2.43	7.53	8.31			11.88	12.70
	Sinapyl alcohol		2.28	2.71	4.72			2.35	5.24
Esters	Geranyl isovalerate							1.77	
	Diisooctyl phthalate			3.40					
Triglycerides	Glycerin	9.61	0.87						
Monoglycerides	2-Palmitoylglycerol	6.14	6.02			60.14			
Aromatic hydrocarbon	p-Xylene						8.35	3.78	6.25

In addition, Table 2 shows that except for common extractives, such as fatty acids and alkanes, the composition of the solvent extractives of PS from hardwood was not as rich as the solvent extractives of the other three herbaceous plants. In general, plant epidermal waxes consist mainly of a mixture of aliphatic hydrocarbons and their derivatives with a carbon chain length between 20 and 40 [24]. The content and composition of aliphatic extractives of agricultural residues from different sources are also different due to their different contents of wax, fat, pigment, and other substances. From Table 1, we found that the content of poplar extractives is less than the other three gramineous materials, which is related to the characteristics of the wood itself, and the extractives of wood are relatively less. Besides, it was confirmed by GC–MS analysis (Table 2) that the types of chemical components in benzene–ethanol extractives and ethanol extractives of CC, SB, WS and PS were generally consistent. It mainly includes fatty acids, fatty alcohols, alkanes, aldehydes, ketones, steroid ketones, sterols, phenols, esters, triglycerides, monoglycerides, aromatic hydrocarbon, etc. However, it can be seen from Table 3 that the component types of the extractives were related to the types of extractants. The benzene–ethanol extractives were mainly fatty acids, while the ethanol extractives were mostly long-chain alkanes. Moreover, the components of ethanol extractives were more abundant than those of benzene–ethanol extractives, which was consistent with previous reports. We speculate that this was due to the greater polarity of ethanol, which could extract more polar compounds [38].

**Table 3:** Contents of main components in agricultural residues

Materials	Glucan/%	Xylan/%	Araban/%	Acid-soluble lignin/%	Acid-insoluble lignin/%
CC	32.86 ± 1.83	31.53 ± 0.01	4.68 ± 0.24	4.42 ± 0.01	15.86 ± 0.10
SB	36.59 ± 0.09	23.74 ± 0.48	4.62 ± 0.47	2.96 ± 0.19	16.98 ± 0.51
WS	35.24 ± 1.07	22.97 ± 0.52	4.08 ± 0.73	3.18 ± 0.04	18.67 ± 1.48
PS	40.07 ± 0.46	16.99 ± 0.17	/	2.66 ± 0.05	23.06 ± 0.76

In summary, the composition of benzene–ethanol extractives was relatively simple, mainly fatty acids, along with a small number of aldehydes, ketones, esters, monoglycerides, and triglycerides. On the contrary, ethanol extractives were complex in composition, were rich in alkanes compared with fatty acids, and included aldehydes, ketones, steroid ketones, sterols, esters, etc. Overall, both BEE and EE confirmed the diversity of plant cuticle wax, which was deposited on the plant surface and tissue interior to protect the plant from biotic or abiotic stress [39]. In other words, these aliphatic extractives were possible obstacles to lignocellulosic biorefining.

### Effects of extraction on the XOS yield of agricultural residues

**Fig. 1:**
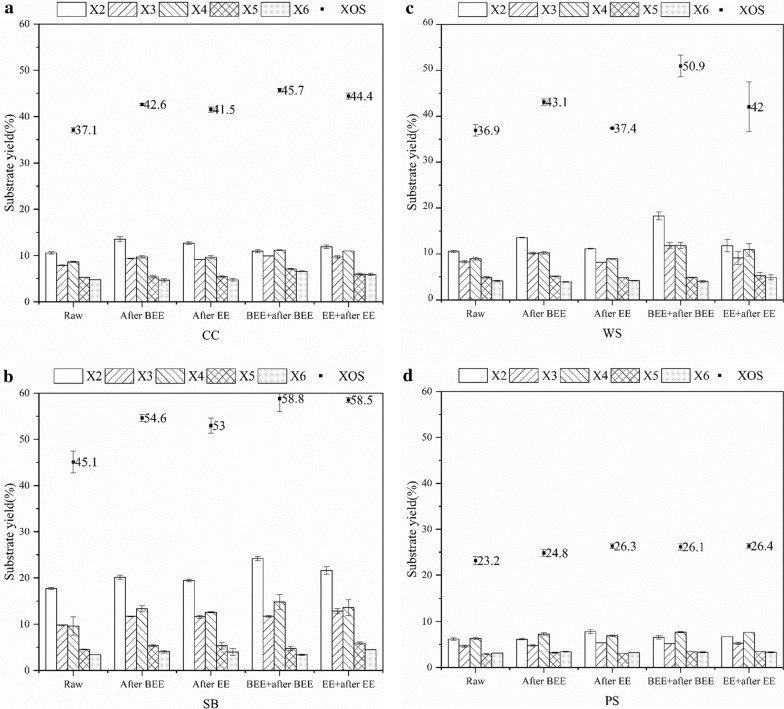
The comparison of the yields of XOS of four agricultural residues under three conditions: (i) acidolysis of raw materials with acetic acid; (ii) acidolysis of the materials after BEE or EE with acetic acid; (iii) acetic acid acidolysis after the re-addition of extractives. **a** Corncob; **b** sugarcane bagasse; **c** wheat straw; **d** poplar sawdust

We studied the conversion of xylan from agricultural residues to verify the effect of organic solvent extraction on the biorefinery of agricultural residues. Next, CC, SB, WS, and PS underwent the same treatment to further evaluate the universal applications of organic solvent extraction and to compare the differences of different agricultural residues affected by their respective extractives. Figure 1a–d shows the results of the degradation of xylan in unextracted agricultural residues to XOS. After acetic acid catalysis, the XOS yield of CC, SB, WS, and PS was 37.11%, 45.12%, 36.92%, and 23.16%, respectively. The main XOS obtained from the four agricultural residues were xylobiose, xylotriose, and xylotetraose, while xylopentose and xylohexose accounted for the minor components. Next, four types of agricultural residues after BEE and EE were also catalyzed using acetic acid under the same reaction conditions. Figure 1a–d shows the yields and components of the obtained XOS. All four agricultural residues showed an improved yield of XOS, after BEE or EE. Compared with the XOS yield obtained from raw materials without extraction catalyzed by acetic acid, the relative increase of XOS of CC, SB, WS, and PS after BEE catalyzed by acetic acid was 14.79, 21.05, 16.68, and 7.26%, respectively. Similarly, after EE, the relative increase of XOS from acetic acid catalyzed CC, SB, WS, and PS were 11.88, 17.43, 1.26, and 13.64%, respectively. For WS, the wax content was obviously the most. Since the components of plant cuticle wax were mainly non-polar components, we believe that the non-polar solvent benzene has a better wax solubility than ethanol, so BEE was more suitable than EE to improve the degradation ability of WS to acetic acid. Furthermore, in each dataset, the increase in the yield of XOS was also reflected in the content of xylobiose, xylotriose, and xylotetraose, among which xylobiose showed the highest increase, while the change in xylopentose and xylohexose was insignificant.

Thus, the extraction of agricultural residues with organic solvents had a selective and positive effect on the yield of XOS from agricultural residues catalyzed by acetic acid. Generally, SB showed the highest XOS yield among all four agricultural residues, which was since SB was mechanically squeezed before collection, which made it very soft and absorbent, and removed some water extractives, while CC and WS were directly used for pretreatment after air drying, and PS showed very inert xylan conversion since it is derived from hardwood. Both BEE and EE could effectively extract cuticular wax components (aliphatic compounds) from all four agricultural residues. The role of physical and chemical properties of plant cuticular wax in protecting plants, reducing the deposition of dust, pollen, and air pollutants on the surface of plants, in preventing bacterial or fungal invasion is well known [34]. Thus, we assumed that the cuticular plant wax had a similar defense against acetic acid. When BEE or EE was not performed, the cuticular wax resisted the protons of acetic acid in all four agricultural residues and thus may have affected the diffusion of products such as XOS as well as restricted the movement of bacteria or fungi. However, the use of benzene–ethanol or ethanol resulted in partial removal of the cuticle wax components in the agricultural residues. Therefore, the protons of acetic acid were easier to attack the agricultural residues in this case, which accelerated the degradation of xylan and improved the yield of XOS.

The extractives from agricultural residues were extremely complex components. The benzene–ethanol extractives and ethanol extractives were re-added to the corresponding extracted agricultural residues, and the mixture was subjected to catalysis with acetic acid under the previous reaction conditions to study the influence mechanism of BEE and EE in the preparation of XOS from the four agricultural residues catalyzed by acetic acid. Figure 1a–d shows that after the re-addition of benzene–ethanol extractives followed by acetic acid catalysis, the XOS yields of CC, SB, WS, and PS were 45.70, 58.83, 50.92, and 26.14%, respectively. Compared with acetic acid catalyzed raw materials, the relative increase of XOS of CC, SB, WS, and PS after the re-addition of benzene–ethanol extractives were 23.13, 30.39% 37.91, and 12.89%, respectively. Similarly, after the re-addition of ethanol extractives followed by acetic acid catalysis, the XOS yields of CC, SB, WS, and PS were also significantly increased. The yields of XOS in CC, SB, WS, and PS were harvested at 44.42, 58.5, 42.04, and 26.37%, respectively. Compared with the XOS yields obtained from the raw materials, the relative increase of XOS of CC, SB, WS and PS after the re-addition of ethanol extractives were 19.68, 29.66, 13.85 and 13.88%. The results of GC–MS analysis showed that there were more non-polar alkanes in PS extractives. Whether natural or re-added, these components will lead to the reduction of hydrogen proton solubility and transmission capacity of acetic acid dissociation. Therefore, PS has always been the stuff with the worst catalytic performance for acetic acid, while three herbaceous plants have shown the superior catalytic performance of acetic acid.

Thus, the re-addition of benzene–ethanol extractives or ethanol extractives to the extracted agricultural residues promoted the degradation of xylan into XOS. The components of the extractives not only contained aliphatic compounds, such as fatty acids, aldehydes, and alkanes, but also included some oligosaccharide substances, based on the results of NREL-HPLC analysis, as listed in Table 1. Therefore, the effective conversion of saccharides contained in the extractives during the acetic acid catalysis process may be one of the reasons for increasing the yield of XOS, and there may be substances with equivalent acid catalysis effect in the extractive, which may be the main reason for the improvement of XOS yield.

### Extraction effect on xylan degraded by-product

**Fig. 2:**
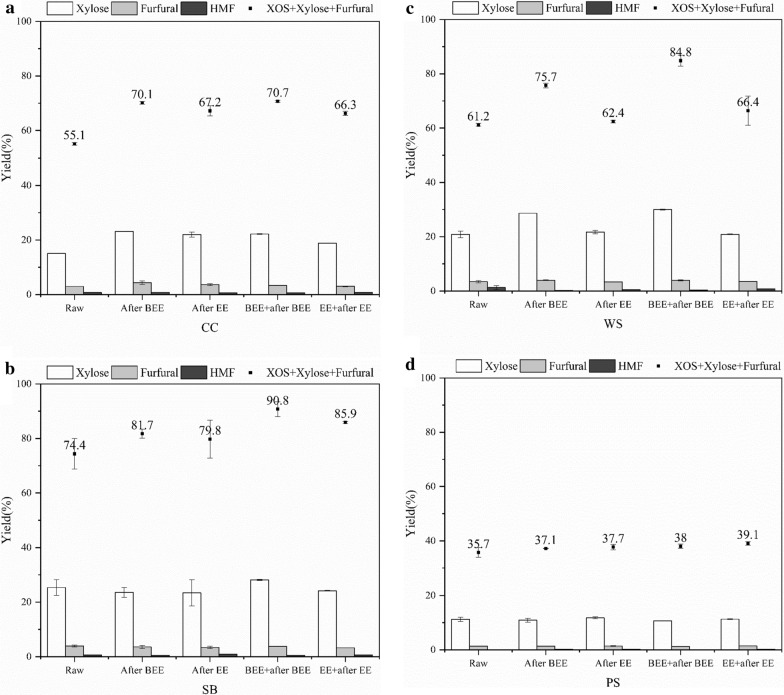
Distribution of by-products components during the catalysis of various agricultural residues with acetic acid: **a** corncob; **b** sugarcane bagasse; **c** wheat straw; **d** poplar sawdust

During the acetic acid catalysis of agricultural residues, excessive degradation of pentose produces furfural and acetic acid [40], and acid-catalyzed dehydration of hexose leads to the formation of hydroxymethyl furfural [41]. These substances, which are the by-products of lignocellulosic biorefining, affect the industrial value of the main products. However, they also have important roles, such as furfural and hydroxymethyl furfural are precursors of synthetic commercial chemicals and liquid fuels [42]. Here, we investigated the degradation mechanism of xylose, furfural, and hydroxymethyl furfural, which were by-products produced in the process of preparing XOS from four agricultural residues. As shown in Fig. 2a–d, before and after organic solvent extraction, CC, SB, WS, and PS were catalyzed by acetic acid to obtain xylose, furfural, and hydroxymethyl furfural as by-products, along with high value-added XOS. Their degradation pattern was consistent with the variation in the yield of XOS. Before and after extraction, the yield of xylose from CC, SB, WS, and PS catalyzed by acetic acid was in the range of 15.05–23.13%, 27.61–32.36%, 20.85–29.97%, and 10.64–11.77%, respectively. When four agriculture residues, including corncob, sugar bagasse, wheat straw, and poplar sawdust, were pretreated with acetic acid, the yield of XOS obtained from poplar was the lowest. The main reason might be due to the inerter chemical structure, lower xylan content and higher lignin content in woody materials that agricultural residues [20]. The xylose and XOS produced from the four agricultural residues catalyzed by acetic acid before and after BEE or EE maintained a reasonably constant proportion, and the content was within the normal range. In several groups of experiments, the content changes in furfural and hydroxymethyl furfural were insignificant. In other words, CC, SB, WS and PS were subjected to BEE and EE, respectively, and then catalyzed with acetic acid, which improved their XOS yields, but a large number of by-products such as furfural and hydroxymethyl furfural were not accumulated under these conditions. Therefore, this confirmed that BEE and EE increased the accessibility of agricultural residues and their acidolysis selectivity. Among the four agricultural residues, CC, SB, and WS, from gramineous plants, showed better acidolysis and extraction applicability compared with PS, which is derived from woody plants. Figure 1d shows that the yield of XOS from acetic acid catalysis of PS also improved after extraction. However, the hardwood characteristics of PS caused an inertness to degradation of poplar xylan, which put PS at a disadvantage in the preparation process of XOS.

The total yield of the XOS + xylose + furfural (XXF) was used as an evaluation index to show the effect of extraction more intuitively on the degradation and dissolution of xylan components of these agricultural residues, which also summarized the conversion efficiency of the xylan components of these agricultural residues catalyzed by acetic acid before and after BEE and EE. As shown in Fig. 2a–d, after BEE and EE, the maximum XXF of CC, SB, WS, and PS was 70.71, 90.75, 84.84, and 39.08%, respectively, while the XXF of CC, SB, WS, and PS only catalyzed by acetic acid was 55.13, 74.36, 61.19, and 35.70%. Consequently, the solvent extraction of agricultural residues intensified the hydrolysis of xylan, which improved the acetic acid catalytic efficiency of xylan.

### Effect of extraction on the enzymatic hydrolysis of cellulose

**Fig. 3:**
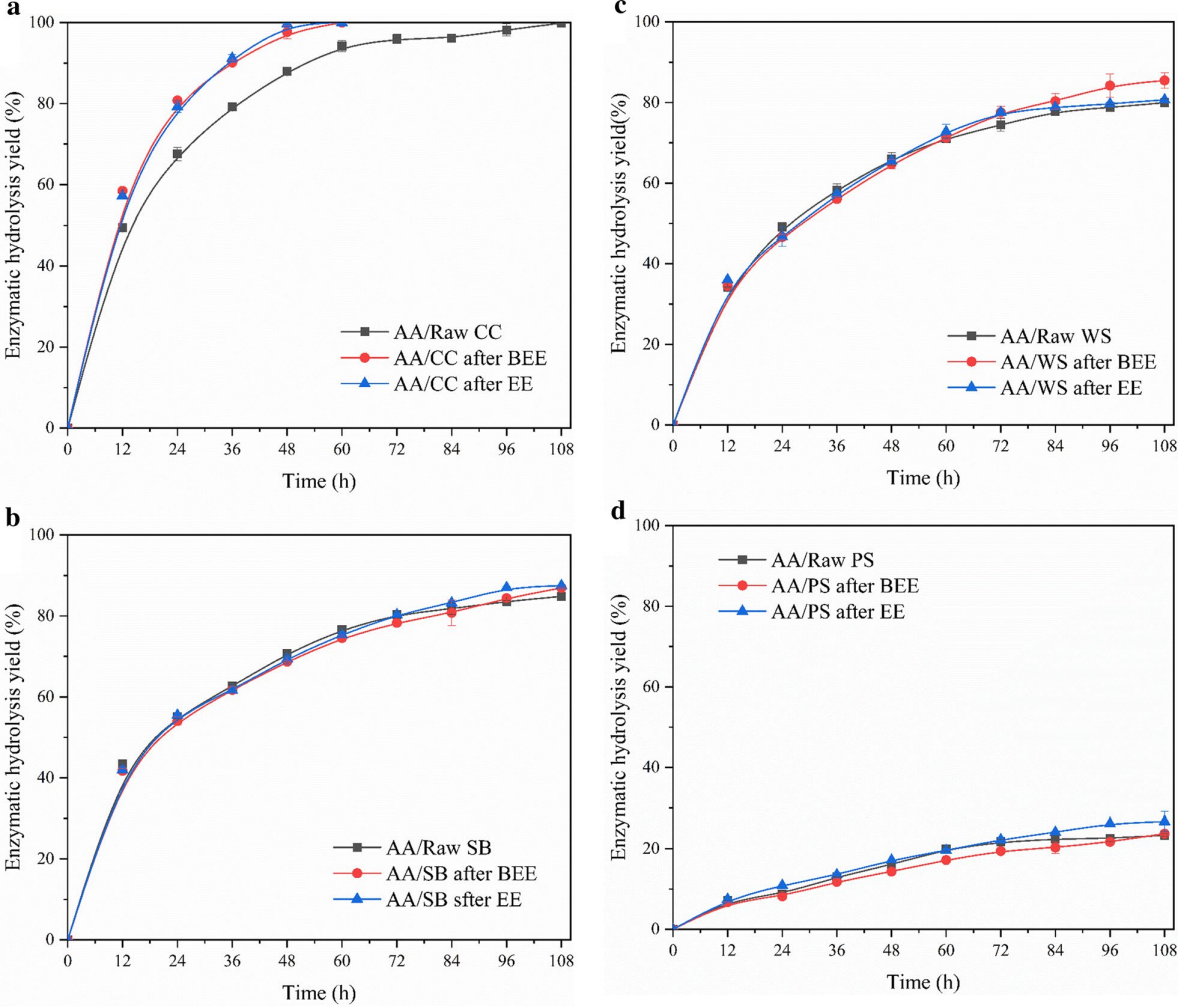
Enzymatic hydrolysis of solid residues treated with acetic acid from various agricultural residues before and after extraction. **a** Corncob; **b** sugarcane bagasse;** c** wheat straw; **d** poplar sawdust

Hemicellulose is generally considered as one of the important physical barriers for enzymatic hydrolysis of cellulose [43]. During the previous stage of acetic acid catalysis, most of the hemicellulose xylan components from CC, SB, WS, and PS were removed. We enzymatically hydrolyzed the solid residues of acetic acid treatment of the four agricultural residues, before and after extraction using cellulase to study the impact of BEE and EE on enzymatic hydrolyzability of cellulose. Figure 3a–d shows the enzymatic hydrolysis yields of the four agricultural residues. For CC, SB, WS, and PS without extraction, the maximum enzymatic hydrolysis yields were 99.90, 84.84, 79.96, and 23.34%, respectively, after 108 h of enzymatic hydrolysis of the acetic acid-catalyzed solid residues. However, the acetic acid-catalyzed solid residues of CC, SB, WS, and PS after BEE obtained the highest enzymatic hydrolysis yields of 100, 86.92, 85.48, and 23.73%, respectively, within 108 h of enzymatic hydrolysis. In simple terms, the removal of aliphatic compounds from four agricultural residues by BEE slightly improved the enzymatic hydrolyzability of the corresponding solid residues. Additionally, due to the differences in lignin content and structural characteristics between herbaceous plants and woody plants, the enzymatic hydrolyzability of poplar was much worse than that of the three gramineous plants, i.e., CC, SB, and WS. The maximum enzymatic hydrolysis yields of acetic acid-catalyzed solid residues from CC, SB, WS, and PS after EE was 100, 87.51, 80.66, and 26.71%, respectively, after enzymatic hydrolysis for 108 h. Similarly, the enzymatic hydrolyzability of materials after EE also improved compared with the raw materials without extraction. As can be seen from Fig. 3a, although the improvement in the enzymatic hydrolyzability of the four agricultural residues by BEE and EE was not obvious, the improvement of the enzymatic hydrolyzability of the CC by solvent extraction could be relatively prominent among the four agricultural residues. The enzymatic hydrolysis yield of acetic acid-catalyzed solid residues from CC after BEE or EE and 60 h of enzymatic hydrolysis was the same as that obtained after 108 h of enzymatic hydrolysis. The increase in enzymatic hydrolysis rate of CC was not only due to the crystallinity and specific surface area of cellulose itself [44], but also due to the largest increase in XXF of CC after BEE or EE (as shown in Fig. 2a). This indicated that CC had the highest increase in xylan dissolution rate, which indirectly improved the enzymatic hydrolyzability of cellulose from CC.

However, there was no significant improvement in the enzymatic hydrolyzability of subsequent solid residues after BEE and EE of agricultural residues. As previously described, the surface wax components of the agricultural residues were effectively extracted by BEE and EE, which increased the accessibility of hemicellulose. However, the enzymatic hydrolyzability of cellulose was affected by a variety of physical and chemical factors, and lignin was considered as one of the main obstacles to the inertness of enzymatic hydrolysis of cellulose [42, 45, 46], while BEE and EE could only partially remove them. Therefore, the extraction and dissolution of aliphatic compounds mainly affected the directional degradation of xylan, but has no obvious effect on the enzymatic hydrolyzability of cellulose.

### Mass balance calculation of EE technology in biorefinery of agricultural residues

**Fig. 4:**
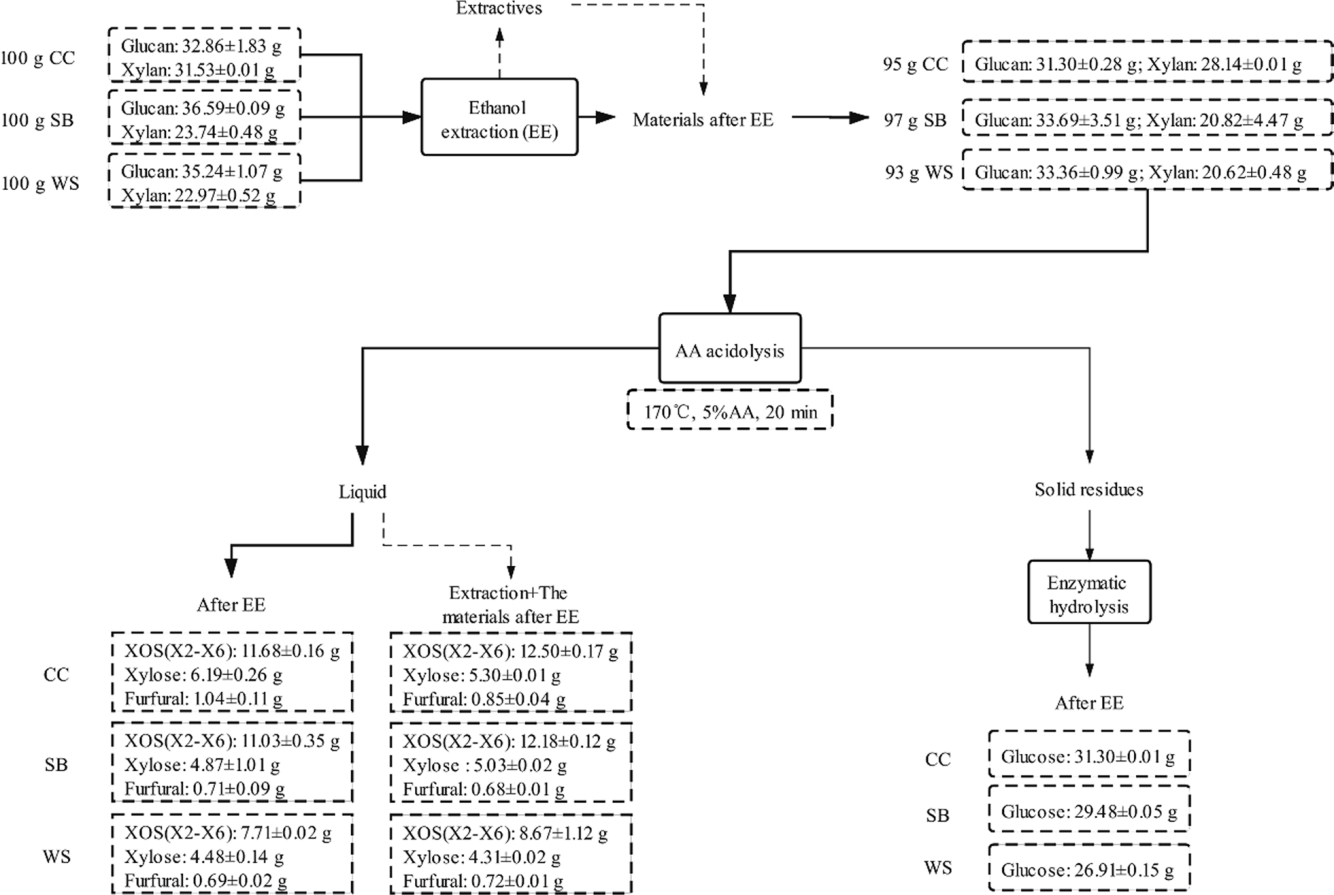
Comparison of the mass balance of the products of acetic acid acidolysis and enzymatic hydrolysis from the agricultural residues after EE

Overall, BEE and EE had a greater positive impact on CC, SB, and WS compared with PS. For WS with more wax, BEE was more effective than EE alone in increasing the XOS yield. Therefore, solvent extraction to remove the aliphatic compounds could promote the biorefinery of agricultural residues. However, benzene is a carcinogenic toxic substance, which is not suitable for producing edible XOS. Moreover, the addition of benzene to ethanol increased the cost of the extraction solvent. Thus, it was more reasonable to choose EE as an auxiliary treatment method for the biorefinery of grass materials while maintaining a balance of efficiency, safety, and cost. Next, we performed a comprehensive evaluation of CC, SB and WS after EE. The xylan degradability and cellulose enzyme hydrolyzability of the agricultural residues before and after extraction of the aliphatic compounds were taken as evaluation indexes. Mass balance was based on 100 g CC, SB, and WS, respectively. Figure 4 lists the calculation results of each material. CC, SB, and WS were extracted with ethanol and then subjected to acetic acid acidolysis to obtain 11.68, 11.03, and 7.71 g XOS, respectively. The pretreated solid residues were subjected to enzymatic hydrolysis to obtain 31.30, 29.48, and 26.91 g glucose, respectively. When the ethanol extractives were added back to the materials after extraction and mixed for acidolysis with acetic acid, 12.50, 12.18, and 8.67 g XOS were obtained from CC, SB, and WS, respectively. Therefore, it means that EE can effectively optimize the acetic acid degradation rate of xylan in agricultural residues to obtain a high XOS yield. The re-addition of ethanol extractives was also conducive to the dissolution of XOS. The existence of xylose and furfural as two by-products confirmed the high dissolution rate of xylan. At the same time, cellulose was mostly converted into glucose by enzymatic hydrolysis. In summary, EE and the re-addition of extractives provide an auxiliary method for efficiently obtaining high value-added XOS and fermentable monosaccharides from agricultural residues.

## Conclusions

Organic solvent extraction was used to study the effect of aliphatic compounds on the acidolysis and enzymatic hydrolyzability of four agricultural residues. We found that removing the aliphatic compounds effectively improved the xylan degradation ability and had a minor impact on the enzymatic hydrolyzability of cellulose. Organic solvent extraction has a more significant optimization effect on herbaceous plants than on hardwood. EE was an effective and appropriate pretreatment auxiliary method because it was safer and more environmentally friendly than BEE. Overall, organic solvent extraction technology provided a promising direction for the industrial production of XOS from herbaceous xylan.

## Methods

### Materials

CC, WS, and PS were procured from the Jiangsu Province of China, while SB was procured from the Hainan Province of China. All materials were crushed into small particles of 20–80 mesh size by a plant grinder (A 11 basic Analytical mill) and were air-dried for a week to maintain the moisture content below 10%. Next, the contents of glucan, xylan, araban, and lignin in the four typical agricultural residues were determined by following the National Renewable Energy Laboratory method [47], as shown in Table 3.

### Soxhlet extraction of agricultural residues

A Soxhlet extractor (1 L; self-built) was used for extracting four types of agricultural residues using a benzene–ethanol solution (volume ratio 2:1 (v/v)) and an ethanol solution, separately at 135 °C for 6 h [48]. Based on the liquid loading capacity of the extraction equipment, a solid–liquid ratio of 1:37.5 (w/v) was used, and only 20 g of the dry substrate was loaded for each extraction. After extraction, the agricultural residues were placed in a fume hood overnight for evaporation to remove residual organic solvents, and the remaining liquid was stored at room temperature.

### Pretreatment of agricultural residues with acetic acid

The acetic acid pretreatment of agricultural residues was performed in a 30-mL stainless-steel tube (Ф 30 mm × 85.0 mm) and capped with a screw cap. The total reaction solution volume was 15 mL. Using a solid–liquid ratio of 1:10 (w/v), 1.5 g of dry matter was mixed with the diluted acetic acid solution. After soaking for one hour at room temperature, the stainless-steel tube was immersed in an electrothermal thermostatic oil tank (Digital oil bath HH-SA, Jintan Youlian Instrument Research Institute, Changzhou, China) to perform the reaction. The reaction conditions were determined based on the difficulty in processing the poplar material and were based on previous literature. The reaction was carried out at 170 °C for 20 min with an acetic acid concentration of 5% (v/v) [13, 18, 20]. After the reaction was terminated, the stainless-steel reaction tube was immersed in cold water and cooled rapidly, followed by the phase separation of the acetic acid mixture of lignocelluloses. Next, the concentration of xylan degradation products, such as XOS, xylose, furfural, etc., in the liquid was determined, and the solid residue was washed with tap water thoroughly and stored at 4 °C.

### Re-addition of extractives

The organic solvent extraction liquid of the four materials was placed in a fume hood overnight to volatilize and remove the organic solvents to obtain dry solid extractive. The re-addition of the dried solid extractive was also carried out following the process conditions in Sect. 2.3, that is, 1.5 g of dry matter (including dry solid extractive and materials after extraction) and 15 mL 5% (v/v) acetic acid mixed acidolysis for 20 min at 170 °C. According to the Soxhlet extraction ratio of 1:37.5 (w/v), the volume of extraction liquid corresponding to 1.5 g dry matter was determined. After removing the organic solvent in a certain volume of the extraction liquid, the dry solid extractive was collected and re-added to the corresponding extracted samples to perform the above acetic acid pretreatment.

### Enzymatic hydrolysis of solid residues

The solid residues after pretreatment with acetic acid were washed thoroughly with water to remove impurities, such as polysaccharides, monosaccharides, and acids. The solid residues, 0.05 mol/L citrate buffer, and diluted cellulase solution (243.48 FPIU/g and 384.2 mg protein/mL, Cellic CTec2, Novozymes, Sigma Co., Shanghai, China) were mixed at a substrate concentration of 5% (w/v) in a 50-mL centrifuge tube, based on the glucan in the pretreated solids, the enzyme loading concentration of 20 FPIU/g glucan [11]. Tetracycline (0.2% (w/v)) was added to avoid microbial contamination during enzymatic hydrolysis. The enzymatic hydrolysis was performed at 150 rpm for 108 h in a thermostatic oscillator (CLASSIC C24, NEW BRUNSWICK SCIENTIFIC CO., INC. Edison, New Jersey, USA) at pH 4.8 and 50 °C. After the reaction was complete, the enzymatic hydrolysates were centrifuged, and the supernatant was collected to detect the concentration of glucose and cellobiose.

### Analytical method

Chemical compositions of agricultural residues, such as glucan, xylan, araban, and lignin, were measured by following the standard method of NREL [47]. The chemical compositions of the extraction liquids were determined by triple quadrupole gas chromatography–mass spectrometry (GC–MS, Agilent 7000B, Thermo Fisher Scientific Trace ISQ). The constant dry weight of the extractives was determined in a 30 °C oven to obtain a constant weight to prevent the loss of volatile contents. The polysaccharides, monosaccharides, and inhibitors in the dried extractives were detected using NREL-HPLC. The degradation products, such as monosaccharides, furfural, and hydroxymethyl furfural were detected by high-performance liquid chromatography (HPLC, Agilent 1260, USA) using an Aminex Bio-Rad HPX-87H column at a temperature of 55 °C with 0.05 mol/L H_2_SO_4_ as the mobile phase at a flow rate of 0.6 mL/min. Similarly, high-performance anion-exchange chromatography (HPAEC, Dionex ICS-3000, Thermo Fisher, USA) equipped with a CarboPac^TM^PA200 (Thermo Fisher, USA) column was used to detect the composition of XOS (Standard XOS chemicals was purchased from Megazyme Ireland, including xylobiose (X2), xylotriose (X3), xylotetraose (X4), xylopentaose (X5), xylohexaose (X6)) using binary gradient elution with 100 mmol/L NaOH and 500 mmol/L NaOAc as eluents, with the elution procedure presented in Table 4.

**Table 4:** Elution procedures

Retention time	Flow	100 mmol/L NaOH	500 mmol/L NaOAc
min	mL/min	%	%
− 2.3	0.3	100	0
7	0.3	100	0
45	0.3	65	35
50	0.3	65	35
50	0.3	100	0
65	0.3	100	0

The yield of XOS, other degradation products of xylan (xylose, furfural), degradation products of glucan and enzymatic hydrolysis yield were calculated according to Eqs. (1, 2, 3, 4, 5):

1 $$ {\text{XOS yield}}\left( {\text{\% }} \right) = \frac{{{\text{XOS}}\left( {{\text{X}}2 - {\text{X}}6} \right){\text{in the acetic acid hydrolysate}}\left( g \right)}}{{{\text{initial xylan content in raw materials}}\left( g \right)}} \times 100\% , $$

2 $$ {\text{Yield of xylan degradation chemicals}}\left( {\text{\% }} \right) = \frac{{{\text{Degradation chemicals of xylan in the acetic acid hydrolysate}}\left( g \right)}}{{{\text{initial xylan content in raw materials}}\left( g \right)}} \times 100{\text{\% ,}} $$

3 $$ {\text{Yield of glucan degradation chemicals}}\left( {\text{\% }} \right) = \frac{{{\text{Degradation chemicals of glucan in the acetic acid hydrolysate}}\left( g \right)}}{{{\text{initial glucan content in materials}}\left( g \right)}} \times 100{\text{\% ,}} $$

4 $$ {\text{Enzymatic hydrolysis yield}}\left( {\text{\% }} \right) = \frac{{\left( {{\text{Glucose}} + {\text{cellobiose}}} \right){\text{ in enzymatic hydrolysate}}\left( g \right) \times 0.9}}{{{\text{glucan content in solid residue after acetic acid acidolysis}}\left( g \right)}} \times 100{\text{\% ,}} $$

5 $$ {\text{The relative increase of XOS }}\left( {\text{\% }} \right) = \frac{{{\text{XOS yield }}\left( {{\text{BEE or EE or re-addition of extractives}}} \right) - {\text{XOS yield }}\left( {\text{raw material}} \right)}}{{{\text{XOS yield }}\left( {\text{raw material}} \right)}} \times 100\% . $$

## Data Availability

All data generated and analyzed in this study are included in this published article.

## Abbreviations

XOS: Xylo-oligosaccharides
CC: Corncob
WS: Wheat straw
SB: Sugarcane bagasse
PS: Poplar sawdust
BEE: Benzene–ethanol extraction
EE: Ethanol extraction
XXF: XOS + xylose + furfural
